# A potential antibody repertoire diversification mechanism through tyrosine sulfation for biotherapeutics engineering and production

**DOI:** 10.3389/fimmu.2022.1072702

**Published:** 2022-12-08

**Authors:** Xiaotian Zhong, Aaron M. D’Antona

**Affiliations:** BioMedicine Design, Medicinal Sciences, Pfizer Worldwide R&D, Cambridge, MA, United States

**Keywords:** antibody repertoire diversification, tyrosine sulfation, complementarity determination region (CDR), antigen-binding affinity, biotherapeutic engineering and production

## Abstract

The diversity of three hypervariable loops in antibody heavy chain and light chain, termed the complementarity-determining regions (CDRs), defines antibody’s binding affinity and specificity owing to the direct contact between the CDRs and antigens. These CDR regions typically contain tyrosine (Tyr) residues that are known to engage in both nonpolar and pi stacking interaction with antigens through their complementary aromatic ring side chains. Nearly two decades ago, sulfotyrosine residue (sTyr), a negatively charged Tyr formed by Golgi-localized membrane-bound tyrosylprotein sulfotransferases during protein trafficking, were also found in the CDR regions and shown to play an important role in modulating antibody-antigen interaction. This breakthrough finding demonstrated that antibody repertoire could be further diversified through post-translational modifications, in addition to the conventional genetic recombination. This review article summarizes the current advances in the understanding of the Tyr-sulfation modification mechanism and its application in potentiating protein-protein interaction for antibody engineering and production. Challenges and opportunities are also discussed.

## Introduction

Antibody therapeutics represent the largest group of biologic drugs with over 100 approved products on the market, providing a significant treatment impact on cancer, immune-mediated disorders, infectious diseases, and cardiovascular/hemostasis disorders (1, 2). Antibodies have favorable attributes such as metabolic stability, specificity, and potency, which can be further enhanced by protein engineering (3–5). A new wave of multispecific innovation (6–10) as well as recent speedy development of antibody products targeting SARS-CoV-2 (2, 11, 12) have demonstrated antibody therapeutics’ important roles in addressing complex disease pathobiology and global health challenges.

Human immunoglobulin G (IgG) is the dominant antibody drug format for recombinant therapeutic antibodies, among the isotypes of IgA, IgD, IgE and IgM (13, 14). IgGs are heterodimers with two identical heavy chains (HC) and light chains (LC) containing constant domains and variable domains (VH or VL). An intact antibody molecule consists of two fragment antigen binding domains (Fabs) and the fragment crystallizable (Fc) that carries out effector function through binding to different Fc receptor proteins on effector cells or by activating immune mediators like complements (13–16). In the Fab regions, the domains of VL and VH are paired to form the antigen binding site, in which three regions of sequence variability termed the complementarity-determining regions (CDRs) are the hypervariable loops in contact with antigens (13, 14). The paratope at these six CDRs from HC and LC determines antibody’s binding affinity and specificity to the epitope on the antigens.

**Figure 1:**
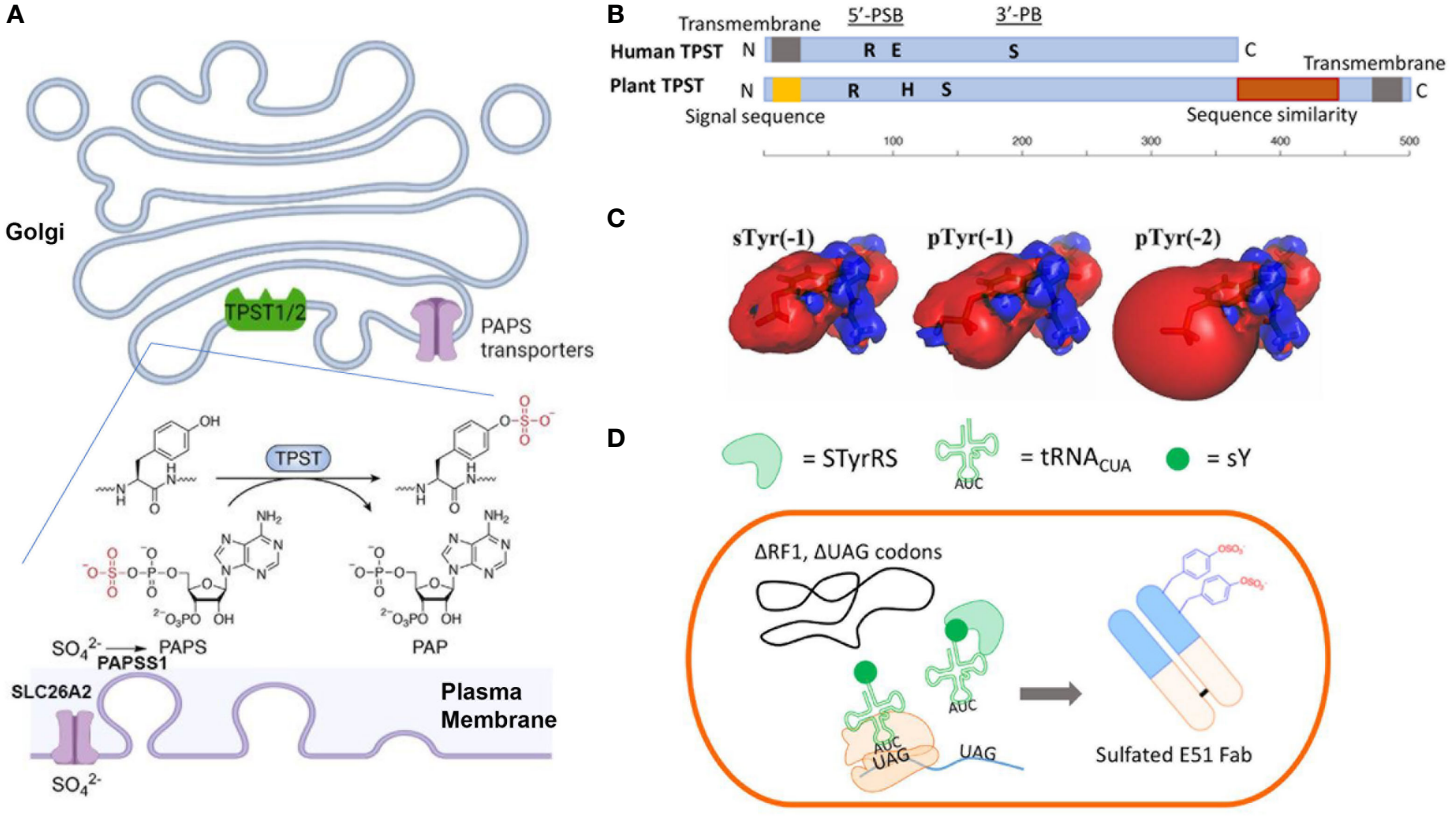
**(A)** Mechanisms of Tyr-sulfation in mammalian cells. **(B)** Primary structures of human TPST and plant TPST (25, 26) (created with BioRender.com). Conserved residue Lys (R) in 5’-PSB, Ser (S) in 3’-PB, and catalytic base residue His (H) and Glu (E) are depicted. **(C)** Comparison of electrostatic potentials for sTyr, pTyr (-1) and pTyr (-2) (27). **(D)** Production of sTyr-containing Fab with extended genetic codon technologies (28).

The diversity of antibody CDRs is mainly generated through the genetic processes of V(variable), D (diversity), and J (joining) recombination and somatic hypermutation, occurred during B cell development in the bone marrow and upon encounters of antigen in the periphery (17–20). The introduction of combinatorial and junctional diversity in the V regions, as well as point mutations in the rearranged V regions after contact of naïve or memory B cells with antigens, create the diversity of antibody repertoires (19–21). Further investigations have revealed that the immune systems can employ post-translational modifications (PTMs) to increase antibody diversification. The first such concrete example is tyrosine (Tyr) sulfation in which a negatively charged sulfo group is added to the phenol group of Tyr through an O^4^-sulfate ester (22–24) (**Figure 1A**). Choe and colleagues first reported that anti-HIV-1 gp120 antibodies’ CDRH3s were Tyr-sulfated that modulated specifically the antibody-antigen interaction (29). This finding demonstrated that Tyr sulfation could be part of the natural humoral immune response to viral infection (30). Subsequently, several other types of unconventional mechanisms which utilize various PTMs to regulate humoral immune responses and antibody diversification have been discovered [reviewed by (31)]. Over the past twenty years, a significant understanding upon the molecular mechanisms of Tyr-sulfation modification and the biological function of the resulting sulfo-Tyr (sTyr) has been shown in protein-protein interaction for peptide hormones, cell surface receptors, virus entry, chemokine signaling, and blood clotting enzymes [see recent reviews, (23–26, 32, 33)]. New examples and mechanistic insights for the role of sTyr in modulating antibody diversification have been reported (28, 34–47). This review focuses on the comprehensive summary of these recent progresses and their potential impacts on applying sTyr in antibody engineering and production. Challenges and perspective for therapeutics development are discussed.

### Tyr-sulfation- a biologically active sulfation

The tyrosylprotein sulfotransferases (TPSTs) pathway (48, 49) is the key synthesis pathway for Tyr-sulfation in antibody. Biological utilization of inorganic sulfate for sTyr, and other sulfated biomolecules (sulfated carbohydrates, or neurotransmitters), requires the metabolic activation into adenosine-5’-phosphosulfate (APS) by ATP sulfurylase (ATPS) and further into 3’-phosphoadenosine-5’-phosphosulfate (PAPS) by APS kinase (22, 23, 25, 26, 32, 33, 50). In animal cells, the APS and ATPS are fused to form a single bifunctional PAPS synthase (PAPSS). The inorganic sulfate ion for the PAPS synthesis is transported from outside into the cytosol through plasma membrane transporter SLC26A2 (**Figure 1A**) (51, 52). Then the PAPS is translocated into the Golgi lumen through the PAPS transporters SLC35B2 and SLC35B3 (53, 54). PAPS is the main activated sulfate group donor for sulfotransferases that catalyze biological sulfation. TPSTs is a major member of sulfotransferases, which also include cytosolic sulfotransferases for sulfation of hormones, neurotransmitters, as well as drugs and xenobiotics (32, 55–57) and membrane-bound carbohydrate sulfotransferase for the sulfation of glycolipid, glycoproteins, and proteoglycans (56, 58). The Golgi-localized membrane-bound TPSTs mediates Tyr-sulfation in antibodies, many other secreted proteins and integral membrane proteins (48, 49, 59, 60).

Tyr sulfation was first reported nearly seventy years ago (61), yet its physiological significance and link to diseases haven’t begun to be appreciated until the cloning of TPST genes more than two decades ago (48, 49). TPSTs are widely expressed in most tissues, and conserved from worm to mammals, and plants but not in yeast and most of the prokaryotes (22, 23, 25, 26). Human TPST1 and 2 exhibit different expression patterns, e.g., TPST1 is more in testis and TPST2 is more in the blood, trachea, thyroid gland, and several other organs (25). These two isoform enzymes also have different substrate specificities (62–64) and distinct mouse knockout phenotypes (65, 66), indicating a difference in specific biological functions (65–67). Animal TPSTs are type II membrane glycoproteins with a length of around 370 amino acids (25, 26). Like those of the cytoplasmic sulfotransferases and saccharide sulfotransferases, the enzymatic domain of TPSTs contains two short sequence motifs, termed 5’-phosphosulfate binding motif (PSB) and 3’-phosphate binding (PB) motif, and a catalytic base residue (His or Glu). They also contain spacing sequences with varying lengths among the functional domains (25, 26, 68) (**Figure 1B**). These enzymes are optimally active at slightly acidic pHs, consistent with their Golgi localization.

There is no unambiguous consensus sequence motif defined for prediction of Tyr sulfation sites in proteins. The common predictors include Tyr residues flanked by acidic residues, large accessible surface areas, and flexible or disordered secondary structure regions fitting well into the cleft of TPSTs. There are several in silico algorithm tools for predicting sulfation, such as the Sulfinator algorithm (69), the SulfoSite (70), The PredSulSite (71), and the Sulfotyrosine site (72). The current publicly available active online tool is Sulfinator (http://web.expasy.org/sulfinator) that has been frequently utilized. Antibodies are underrepresented in these machine learning algorithms, and the data of accuracy in predicting Tyr-sulfation for antibodies remain limited. It is well known that sTyr residues are neighboring with acidic amino acid (73). Presence of acidic residues, glutamic and aspartic acids, within +5 to -5 positions of the Tyr increase the activity of TPSTs (74, 75). These residues make multiple electrotactic interactions proximal to the active site of TPSTs (76–78). A basic residue in the amino-terminal (-1) position of the Tyr abolishes sulfation 76), as the negative charge of glutamic acid at this position is recognized by the backbone amide nitrogen of TSPTs (78). The presence of Gly or Asn residues surrounding sTyr tends to increase sulfation (62). The role of structural flexibility in sulfation regions could imply that the solvent-exposed and flexible CDRs is more likely Tyr-sulfated than the framework regions in antibodies (79).

Through determining protein-protein interaction, Tyr-sulfation has been involved in many biological processes, including blood coagulation, leukocyte rolling, complement cascade, hormonal regulation, viral infection, chemokine signaling, collagen binding, and Wnt signaling (22, 23, 25, 26, 80). These examples in nature illustrate the intricate mechanisms through which sTyr is utilized in protein-protein interactions, providing a guiding principle for the application of sTyr residue [For details see a recent review by (26)].

### Unconventional diversification for antibody repertoire through sTyr.

As a bulky amino acid that can facilitate hydrophobic interaction, Tyr residue is frequently found in CDR regions of antibodies and sulfation can presumably occur to some of these Tyr residues in the CDR loops. Hence discovering sTyr in antibody repertoire shouldn’t be a surprise in principle, yet the finding didn’t happen until 20 years ago. This is probably due to the fact that sTyr is heat labile and can be rapidly hydrolyzed under strong acidic condition. These features made sTyr residue difficult to be identified reliably with analytical tools such as standard Edman sequencing and mass spectrometry (23, 26). The conclusive identification of sTyr residues in antibody CDRs by Choe and colleagues was through traditional radioactive labeling using ^35^S-sulfate (29). A set of patient-derived human monoclonal antibodies against HIV-1 envelope glycoprotein gp120 was metabolically labeled with ^35^S-sulfate in transformed human cell lines during recombinant expression. Only the HCs were labeled and subsequently Tyr residues in CDRH3 were found sulfated. These sTyr residues contribute directly to antibody engagement with gp120, as in the case of CCR5 with overlapping regions in gp120. Despite structural differences between the Fab and CCR5 N-terminus, one sTyr residue in either the Fab or CCR5 is recognized in a similar manner by gp120 (35), indicating that the antibody mimics mechanistically the CCR5 for interacting with gp120. Interestingly, these sulfated antibodies were obtained from three different individuals. sTyr residues in two of these sulfated antibodies were originated from a common V_H_ gene V1-69, whereas those in other three sulfated antibodies were from the longest of six HC joining gene J_H_6. This landmark finding demonstrated for the first time that Tyr-sulfation can contribute to the potency and diversity of human antibody repertoire.

**Figure 2:**
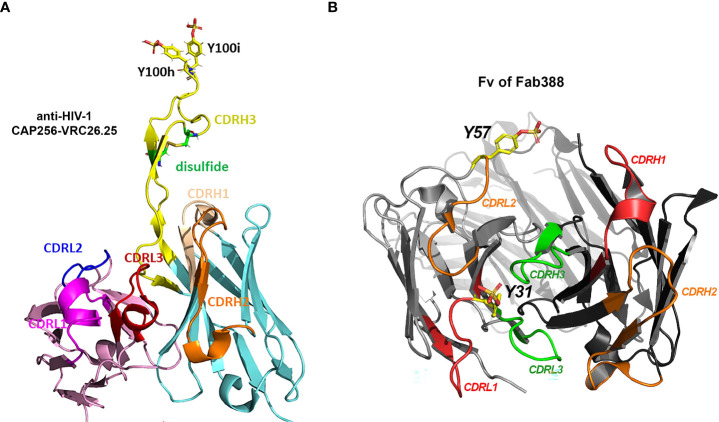
Three types of Tyr sulfation modification at antibody CDRs. **(A)** Structural display of sTyr residues at CDRH3 of anti-HIV-1 antibody CAP256-VRC26.25 (Gorman, Chuang et al. 2020). PDB 5DT1 (47, 81). **(B)** sTyr residues at CDRL1 (Y31) (40) and CDRL2 (Y57) (41) are displayed on antibody Fab structure (Fv of Fab388). Sulfated tyrosine residues were modeled on the corresponding Fab structure PDB 5I1A (14, 82) using Pymol molecular graphics v2.5.4.

There are additional antibodies with sTyr being reported (**Table 1**). Consistent with the knowledge that CDRH3 is much more diverse because of the VDJ gene rearrangement versus V gene only for CDRH1/2, CDRL1/2, and V gene and J gene recombination for CDRL3 (21), there are more sTyr found in the CDRH3. There are a number of examples of clustered sTyr found in CDRH3 (**Table 1**). Some of them were not functional whereas some contributed to the potency and diversity of the antibody. Huang et al. (34) hypothesized that the CDRH3 could be the only CDR long enough and diverse enough to be a substrate for Tyr sulfation. This is further supported by the fact that Tyr is often a bias outcome of the VDJ rearrangement as many IGHD genes have nucleotide sequence TAC (codon for Tyr) at their 3’ end (21), which could further skew toward a potential sTyr site. Interestingly, the non-functional Tyr-sulfation is ordered whereas those functional are disordered and therefore might be flexible enough for stereochemical orientation. This is consistent with the fact that there are quite a few Fab alone structures but no high-resolution structure of functional sTyr-containing Fabs complexed with the target antigens (**Figure 2A**). Regarding the activity, both antibody PG9 and PG16 contained sTyr residue in a unique hammerhead CDRH3 subdomain (**Table 1**), which showed significantly more potency on neutralization than its unsulfated form (36). The specific electrostatic interactions were made between cationic residues of gp120 and sTyr at antibody PG9, as the Tyr sulfate provides a closer match than the standard acidic Asp and Glu side chains (37).

**Table 1:** Summary for sequences and sTyr residues at antibody CDRs.

Antibodies	Amino acid sequences	CDRs	References
412d	.PYPND **Y** ND **Y** APEEGMSWYFDLW(103).	CDRH3	(29, 34)
E51	IAGVAAAGD **Y** AD **Y** DGGYYYDMDVW(103).	CDRH3	(29, 34)
PG9	EAGGPDYRNGYN **YY** DFYDGYYNYHYMDVW(103).	CDRH3	(36)
PG16	EAGGPIWHDDVKY **Y** DFNDGYYNYHYMDVW(103).	CDRH3	(36)
PGT145	HRLRDYFL **Y** NE **Y** GPNYEEWGDYLATLDVW(103).	CDRH3	(37)
2909	DKGDSD **Y** D **Y** NLGYSYFYYMDGW(103).	CDRH3	(37)
CAP256.03	EEWWSD **YY** DFGKQLPCRKSRG-VAGIFDGW(103).	CDRH3	(81)
CAP256.25	EEWWSD **YY** DFGKQLPCAKSRGGLVGIADNW(103).	CDRH3	(81)
Human mAb-Merck	XSXSXD **Y** EGDSD(36)XXXXXXX.	CDRL1	(40)
Human IgG1mAb1-Roche	LIYSASDLD **Y** GVPSR(62).	CDRL2	(41)

Amino acid sequences and sTyr residues at CDRH3 (26, 29, 34, 36, 37, 81), CDRL1 (40), and CDRL2 (41) are listed. Sulfated Tyr residues are in bold and underlined.

CAP256-VRC26.25 (**Figure 2A** and **Table 1**), one of the most potent antibodies targeting V1V2 apex of gp120, contains two sTyr in CDRH3 (43), and uses an extended CDRH3 loop to insert sTyr into the hole at the V1V2 apex. One recent study (47) further indicates that an increased level of Tyr sulfation on this anti-HIV-1 antibody corresponded to more robust antigen binding to the V1V2 domain of gp120 protein. Molecular binding efficacy dropped significantly with a loss of only one sulfo group. Without the sulfation, nearly no binding was detectable. Full sulfation occupancy in the antibody CDRH3 is important for effective high antigen binding and should be regarded as a critical quality attribute for this antibody (47).

Several recent reports provide other examples of Tyr sulfated antibody at the varied CDRs. Different from the modification sites in CDRH3 for anti-HIV-1 antibodies, the Tyr sulfation sites located in the short CDRL1 region (40) or CDRL2 (41) have been recently discovered (**Figure 2B** and **Table 1**). One human antibody produced by stable CHO cells was 40% Tyr-sulfated at its LC CDR1 region (40). The modified Tyr residue (Y31) is surrounded with Glu or Asp residue at -1, +1, +3, +5, supporting its being a substrate for TPSTs. This is the first example in which Tyr-sulfation is on an antibody’s CDRL1 region. During the immunoglobulin rearrangement, CDRL1 contains only V gene fragments. In human germline V-segment loci, there are 51 V_H_ and 70 V_κ/λ_ (83). Therefore it is much less diverse than CDRL3 (with additional J fragment recombination) or CDRH3 (with additional D or J recombination). The biological function of this sTyr is not disclosed in the paper (40), but the fact that this modification was intentionally preserved during stable CHO production suggested its possibly important role in antigen binding (45).

Tyshchuk and colleagues reported that a human IgG1 expressed in stable CHO cells was 21% Tyr-sulfated at its LC CDR-2 region (**Figure 2B** and **Table 1**) (41). The sTyr residue (Y57) is nearby with Asp residue at -1 and -3 position whereas a similar antibody with unsulfated Tyr has a Thr residue at -3 position, supporting the important role of acidic residue on amino-terminal site of Tyr for sulfation. Even though this sTyr is not essential for antigen binding (41), one would expect to see more functional examples of sTyr in antibody CDRs being uncovered, as the functional role of sTyr catches a wider attention.

### Implication of sTyr in antibody engineering

sTyr extends the vocabulary of protein synthesis beyond the standard 20 amino acids (26, 34). It has an important implication in antibody engineering for protein-protein interaction, especially under the fact that Tyr-sulfation is not a rare event, estimated to be ~7% of mammalian proteins and ~1% of all Tyr residues in the eukaryotic proteome (22, 25, 74, 84). sTyr heightens protein-protein interactions *via* two mechanisms. The first one is that Tyr’s aromatic ring engages nonpolar and pi stacking interactions with various binding residues. The second one is that the sulfate group can make electrostatic interactions with positive-charged Arg or Lys residues on the surface of the binding partner. For instance, replacing the sTyr^12^-interacting-Lys^27^ of chemokine CXCL12 with Ala or Glu resulted in 3.9- and 181.7-fold reduction in CXCR4 receptor activation respectively (85).

One advantage of sTyr is that its sulfate ester is anionic at physiological pH, providing an electrostatic component to specific interaction without acting as a base or nucleophile. Tyr sulfate also provides a longer electrostatic arm than the standard acidic Asp and Glu side chains. Sulfate esters are often found in clusters which is an efficient way of generating unique structures to increase interaction affinity. Another advantage of sTyr is that the sulfate ester is stable in physiological conditions. Other than the sulfatases for steroids or carbohydrates (86), no sulfoprotein sulfatase is yet reported for mammalian cells and tissues. sTyr is therefore considered as a long-lasting PTM.

With a nearly identical mass, sTyr and phospho-Tyr (pTyr) has been compared both biologically and biophysically (**Figure 1C**). Both the sulfate group and the phosphate group are PTMs with the addition of an oxoanion functional group (27, 87). Both groups are fully ionized at neutral pH and can increase side-chain polarity. Comparing to pTyr, sTyr makes weaker hydrogen bonds, attributed to the reduced electrostatic potentials (-1 for sTyr vs -2 for pTyr) and smaller dipole moment (27). sTyr sulfate can create distinct ionic contact (88, 89) and provide a unique interaction specificity, even though for some protein-protein-interactions pTyr can partially replace sTyr (90–92). There are cases that pTyr cannot substitute sTyr (93). For CCR7 signaling with CCL21, pTyr can only replace sTyr at position 8 of CCR7 but at postion17 sTyr provides a drastic effect over pTyr (92).

From a protein engineering point of view, sTyr interactions with Arg are weaker significantly than that of pTyr, and even not the same interactions made by the Glu residue. However, sTyr has a greater flexibility than pTyr, attributing to a weaker hydrogen bonding interaction. It provides a unique interaction specificity determinant, as in the example that phosphate can’t replace sulfate for interacting with Lys145 of CCR5 (34). Many secreted proteins contain basic patches on their protein surfaces. Charge-charge interaction between sulfate group and Lys/Arg residues on these proteins might provide a way of affinity up-regulation for Tyr-sulfated antibodies. This mechanism might have been utilized by human antibody repertoire for targeting this kind of antigen.

As a known player for protein-protein interaction, sTyr usually mediates the interactions through relatively short protein segments, e.g., amino-termini of cell surface proteins and receptors. This feature fits ideally with the length of the CDR loops in antibodies, even though not all sTyr are important for protein-interaction, with some being detrimental (28). Interestingly, even a peptide derived from the Tyr-sulfated CDRH3 of E51 antibody is sufficient to engage with virus spike protein and neutralize virus isolates (94, 95). Recently Siguna Mueller wrote an article on rarely-recognized antibody diversification against SARS-CoV and SAR-CoV-2 with a potential role of sulfated antibodies (96), as the basic residue stretches on the surface of the viral spike protein (97) can be targeted by the negatively-charged Tyr-sulfate. More novel examples in protein engineering with sTyr are expected to appear in the literatures.

### Clinical development and manufacturing of sTyr-containing antibodies

Several sTyr-containing antibodies such as PG9, PG16, and CAP256-VCR26 have entered clinical trials or preclinical stages as broadly neutralizing antibodies for the treatment and prevention of HIV infection (98–100). These sTyr-containing antibodies have been successfully produced by stable CHO cells (45, 47) or in plants (38, 44).

A major challenge for Tyr sulfation is the difficulty of expressing target proteins in a homogenously sulfated state. Chemical synthetic approaches have been developed for peptide synthesis, but not for large proteins (101). The genetic code expansion technology (**Figure 1D**) utilizes an engineered tyrosyl-tRNA synthetase/tRNA pair that co-translationally incorporates sTyr into the UAG codons in bacterial (102) and mammalian cells (103, 104). These new methods provide a promising way to incorporate sTyr into proteins site-specifically for evaluating the roles of individual sulfations.

Sulfated antibodies have been routinely produced by mammalian cells, even though different sulfation levels were observed (37, 45). This is likely due to different gene expression in sulfation pathway like those of TPSTs, which can be rescued by TPST overexpression. Ectopic expression of either TPST1 or TPST2 can result in a high percentage of Tyr-sulfated antibodies, yet producing a full Tyr-sulfation occupancy on modified sites remains to be a challenging task (47).

Plant-based expression system has been employed for cost-effective antibody production (38, 44, 46). Plants encode a single TPST with a carboxyl-terminal transmembrane domain which is different from the amino-terminal segment for human TPSTs (**Figure 1B**) (26, 105). Plant TPST has putative 5’-PSB ad 3’-PB motifs even though the similarity with human counterparts is low. It shares additional carboxyl-terminal sequence similarity with heparan sulfate 6-O-sulfotransferase (105). Initial sulfo-antibody expression in plant did not produce detectable sTyr, indicating that mammalian-type of sulfation does not naturally occur in plant (38, 39). When human TPST1 was engineered in plants, a plant Golgi-targeting sequence was needed for targeting the enzyme to late Golgi compartment (38). A drastic improvement for Tyr sulfation was subsequently observed. Several sTyr-containing antibodies have been produced successfully by this engineered plant-based system for anti-viral therapy (44, 44, 46).

### Challenges and perspectives

PTMs like Tyr sulfation in mammalian cells provide *de novo* synthesized proteins with additional level of structural diversity beyond their primary sequences. These PTMs impose new biological functions and activity modulations as well as further physiological consequences. For Tyr sulfation modification, it results in versatile interaction motifs that are critical for numerous high-affinity physiological interactions. It is fascinating that sTyr also has a role in regulating humoral immune responses and contributing to the extent of the antibody diversification as first uncovered nearly two decades ago. This PTM has provided a new strategy for therapeutic antibody engineering and diversification.

However, there are still significant challenges being presented to Tyr sulfation. Specificity determinants for Tyr sulfation remain to be well defined. Better understanding how TPSTs engage with their substrates is essential for evaluating the location and function of sTyr at CDRs. Moreover, feasible and reliable analytical methods for detecting sTyr are needed for understanding many under-documented protein substrates in protein database. Elucidating the biological mechanisms for these newly identified sulfoproteins are challenging, whereas accumulating these physiological knowledges is important for designing new sTyr-containing antibodies and therapeutic proteins. For instance, three sTyr residues (Y46, Y48 & Y51) of P-selectin glycoprotein ligand-1 (PSGL-1) make ionic interactions with protonated His residues (H154, H153, & H100) of the PSGL-1 ligand VISTA in acidic tumor microenvironments, which is disrupted by the imidazole sidechain deprotonation at the physiological pH7.4 (106). This finding reveals a novel role of sulfation in a pH-selective binding interaction for future protein engineering design.

Developing new technologies in producing site-specific sulfo-antibodies is helpful in determining the structure-function relationship. While the genetic codon expanding technology is promising, its bioprocessing bottlenecks need to be overcome for a large-scale production. In addition, producing sTyr-containing secreted full-length intact antibody proteins with this methodology remains to be demonstrated. Further understanding of sTyr as a critical drug product attribute is also warranted for sulfo-biotherapeutics. sTyr is known to work together with N- or O-glycans (24), which are also present at CDRs for enhancing antigen binding (107, 108). With more knowledge being gathered for the roles of these PTMs at CDRs, one expects molecular insights and mechanisms of these unconventional strategies for antibody diversification will be further revealed.

## Data availability statement

The original contributions presented in the study are included in the article/supplementary material. Further inquiries can be directed to the corresponding authors.

## Author contributions

XZ and AD conceptualized, wrote, and edited the manuscript. All authors contributed to the article and approved the submitted version.
